# Pontocerebellar Hypoplasia Diagnosed on Autopsy: A Case Report

**DOI:** 10.7759/cureus.8178

**Published:** 2020-05-18

**Authors:** George S Stoyanov, Emran Lyutfi, Deyan L Dzhenkov, Lilyana Petkova

**Affiliations:** 1 General and Clinical Pathology/Forensic Medicine and Deontology, Medical University of Varna, Varna, BGR; 2 Neurology and Neuroscience, Medical University of Varna, Varna, BGR

**Keywords:** pontocerebellar hypoplasia, cerebellum, histology, morphology

## Abstract

Pontocerebellar hypoplasia (PCH) is a diverse group of autosomal recessive genetic conditions presenting with hypoplastic changes in the brainstem, cerebellum, and spinal cord. It clinically manifests with neurological symptoms, respiratory failure, and often in a combination with other malformations of the internal organs and musculoskeletal system. In this report, we present an autopsy case report of a two-month-old female patient with blood-relative parents. The patient presented clinically with neonatal-onset respiratory failure, mild neurological symptoms, facial dysmorphism, and developmental delay. On autopsy, the cerebellum and brainstem were severely hypoplastic, and the diagnosis of PCH was established grossly. The central nervous system (CNS) revealed specific hypoplastic changes in the structures, with a decreased neuronal count, stratification disturbances of the cortex of the cerebellum, and cellular misarrangement. The morphological findings in the CNS and their associated parenchymal organ changes, even in the absence of a genetic test, were specific enough to identify PCH type 1B as the main condition.

## Introduction

Pontocerebellar hypoplasia (PCH) is a diverse group of conditions associated with hypoplastic changes in the cerebellum, brainstem, and spinal cord. it results in respiratory failure and a myriad of neurological changes, such as hydrocephalus, microcephaly, seizures, and overall developmental delay [1,2]. There are 10 identified types of PCH, each with its associated genetic mutations and clinical spectrum of changes. Some types of PCH have additional subtypes based on minor deviations of the clinical spectrum and different genetic changes [1,3].

Clinically, PCHs are most commonly diagnosed based on the specific presentation of the patient, together with imaging modalities of the central nervous system (CNS): CT or MRI and genetic tests [4]. The gross morphological changes observed during autopsy include changes in the size, shape, and foliation of the cerebellum, with decreased cerebellar weight [5]. Histology usually reveals changes in the stratification of the cerebellar cortex, foliation changes, decreased neuronal count in the nuclei of the cerebellum and brainstem, as well as changes in the neuronal count of the anterior horns of the spinal cord, depending on the type of PCH [4,5].

## Case presentation

A two-month-old female patient, transferred from a tertiary healthcare center, was referred for autopsy with signs of developmental delay and respiratory failure. The patient had a medical history of being the fifth birth out of six overall pregnancies, with the parents being blood relatives (second cousins). The mother had suffered from an acute respiratory infection (unspecified) in the seventh gestational month and was a heavy smoker. The patient had been delivered 10 days prematurely per via naturalis with a weight of 2,250 g, height of 48 cm, and an Apgar score of 6 on the first minute and 8 on the fifth minute.

On the second day postpartum, decreased reflexes and generalized muscular hypotension had been noted. Following 24 days in the neonatal intensive care unit, the patient had been de-hospitalized. Two weeks later, the patient had been hospitalized again after rejecting food, with the presence of a severe dry cough and muscle hypotension. Upon admission, no fever had been noted; radiology had shown lung consolidation, and the patient had been treated with antibiotics for bilateral pneumonia. After two weeks of treatment, the condition of the patient had not improved, and she had been referred to our hospital for further diagnostic tests and treatment. However, upon admission, the patient's condition was extremely poor and a lethal exit was registered, following extensive reanimation. The patient was referred for an autopsy to identify the underlying condition.

**Figure 1:**
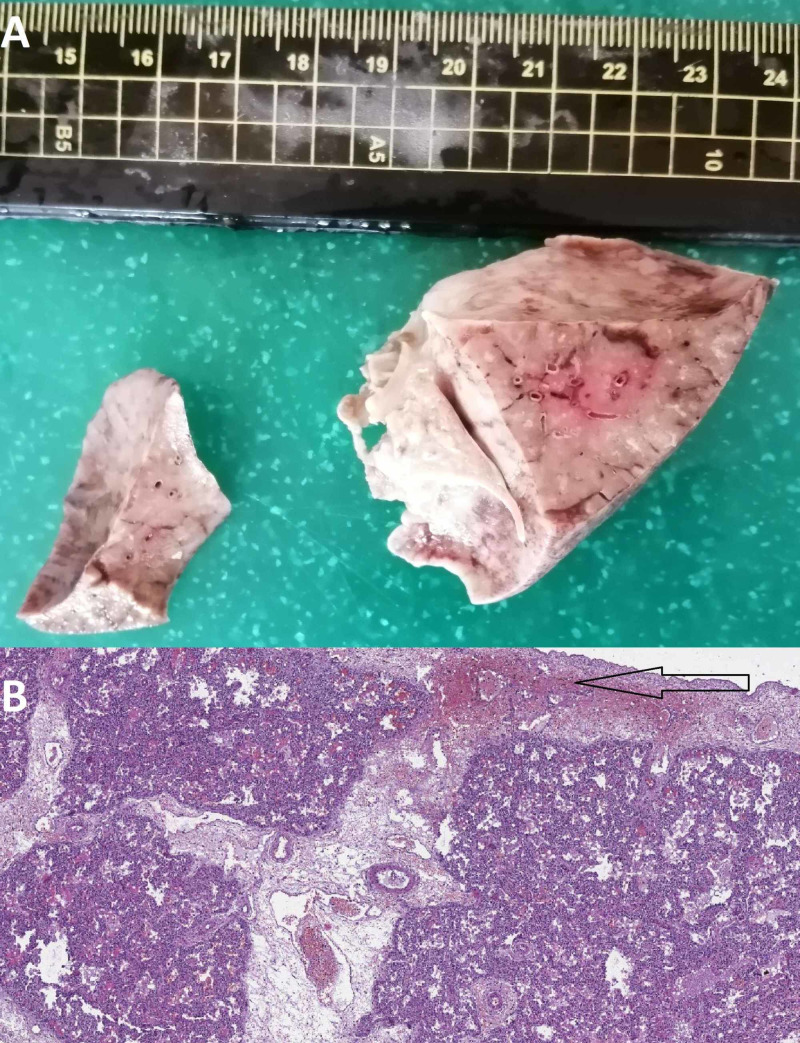
Gross and histological findings in the lung A: gross view of the consolidated lungs; B: histology showing diffuse subtotal atelectasis and a subpleural hemorrhage (arrow) (hematoxylin and eosin stain, original magnification x40)

Before autopsy, the patient weighed 3,140 g, was 50 cm in height, and head circumference was 33 cm (microcephaly). Furthermore, mild facial dysmorphism was noted, with retrognathia, gothic palate, and bulged zygomatic bones. On section of the thorax, the lungs were deformed and retracted towards the hilus with subpleural hemorrhages. The left lung weighed 30 g and the right one 38 g. A floating probe was performed with both lungs sinking. On a section floating probe, only the upper lobes of the lungs were semi-emergent in the liquid, with both inferior lobes and the middle lobe of the right lung sinking. On cross-section, the lungs were airless and consolidated. Histology revealed subtotal atelectasis and subpleural hemorrhages (Figure 1).

**Figure 2:**
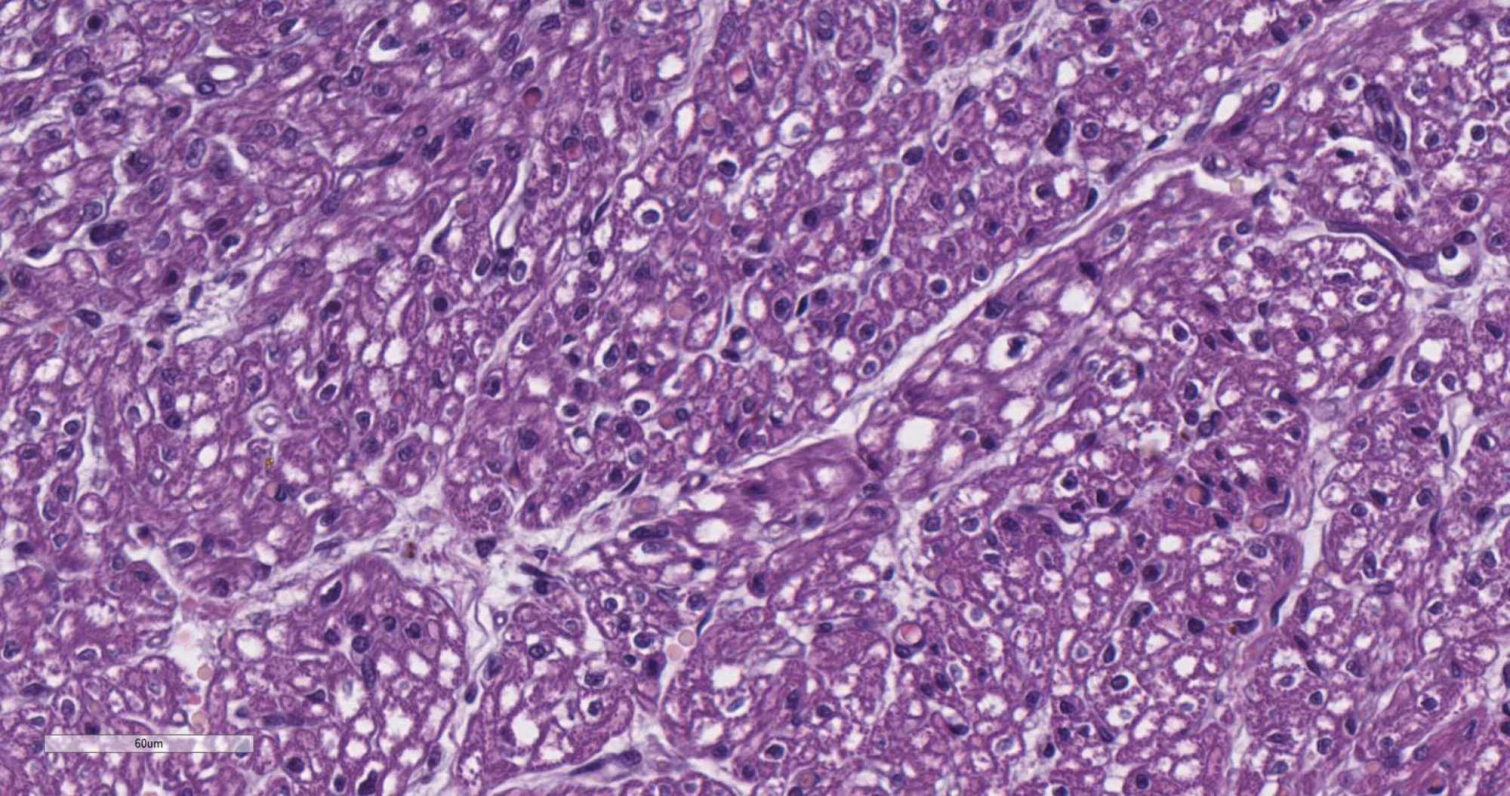
Diffuse granular degeneration of cardiomyocytes (hematoxylin and eosin stain, original magnification x400)

The atria of the hearth were dilated, and the cardiac weight was 28 g. On section, a persistent and dilated foramen ovale was observed. Histology revealed a granular change in the cardiomyocytes, predominantly in the atria (Figure 2).

**Figure 3:**
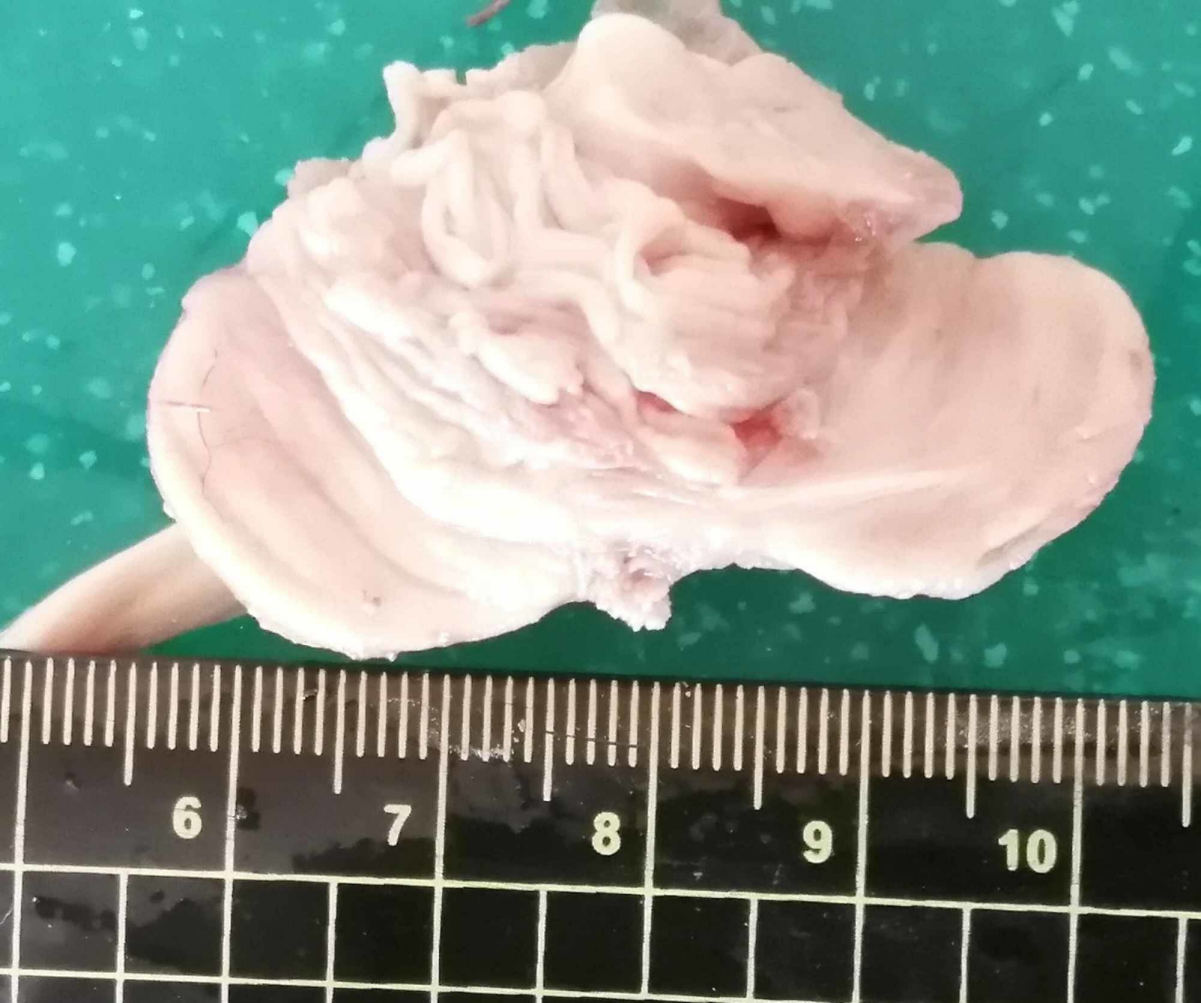
Gross view of the cerebellum

**Figure 4:**
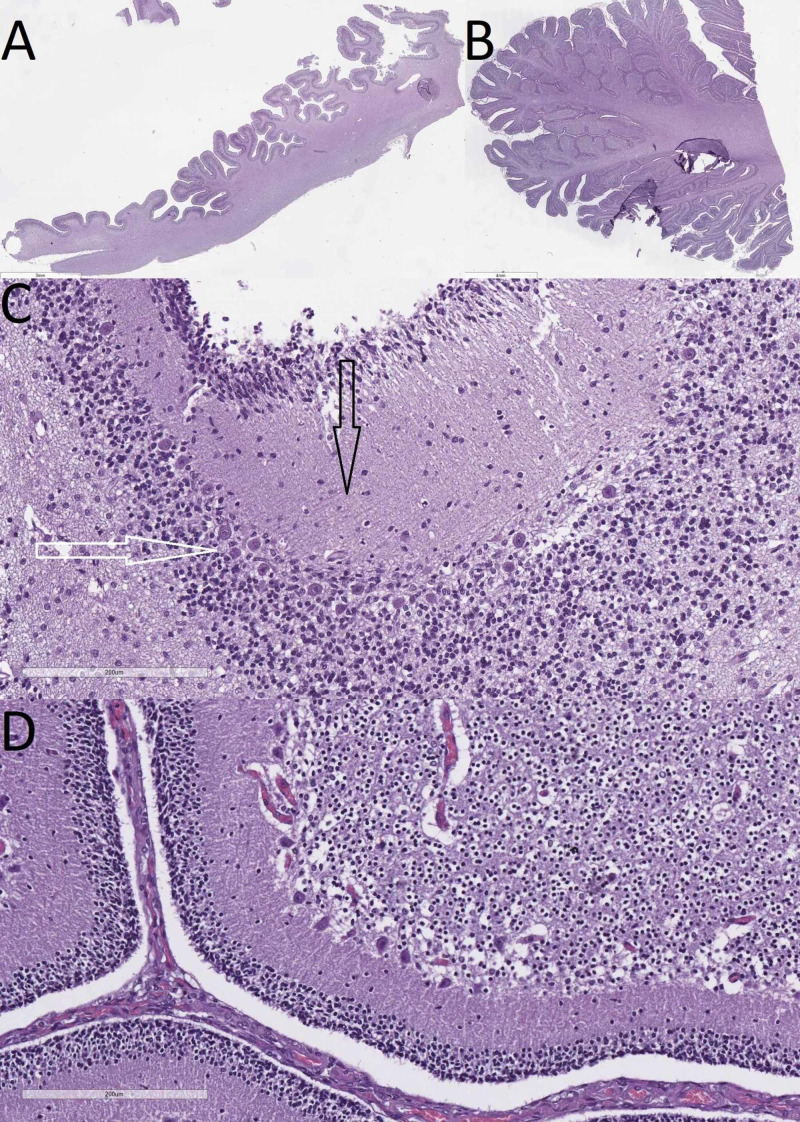
Comparison between the cerebellum of the case and age-adjusted control A: macro slide of the cerebellum from the case (hematoxylin and eosin stain); B: macro slide view of the cerebellum from healthy, age-adjusted control (hematoxylin and eosin stain); C: cortical architecture from the case with tangential Purkinje branching (black arrow), Purkinje cell disarrangements (white arrow), and varying cortical layer thickness (hematoxylin and eosin, original magnification x200); D: cortical architecture from healthy, age-adjusted control (hematoxylin and eosin, original magnification x200)

Cranial section showed undisturbed dural duplicatures. Following the section of the tentorium cerebelli and the extraction of the cerebrum, cerebellum, brainstem, and spinal cord, the brainstem and cerebellum were noted to be severely hypoplastic, with a lack of basal foliation of the cerebellar cortex. The combined weight of the cerebrum, cerebellum, brainstem, and spinal cord was 270 g, with the cerebellum weighing only 5 g (Figure 3). On section of the CNS, the lateral ventricles were noted to be dilated, especially the occipital horns. Histology of the cerebellum revealed subtotal lack of tertiary foliations, large sections of only secondary and primary foliations, abortive mushroom-like foliations, complete lack of foliations on the basal surface, tangential Purkinje branching, disarrangement of the Purkinje cells, and a severe variation of the cortical layer thickness and neuronal count (Figure 4).

**Figure 5:**
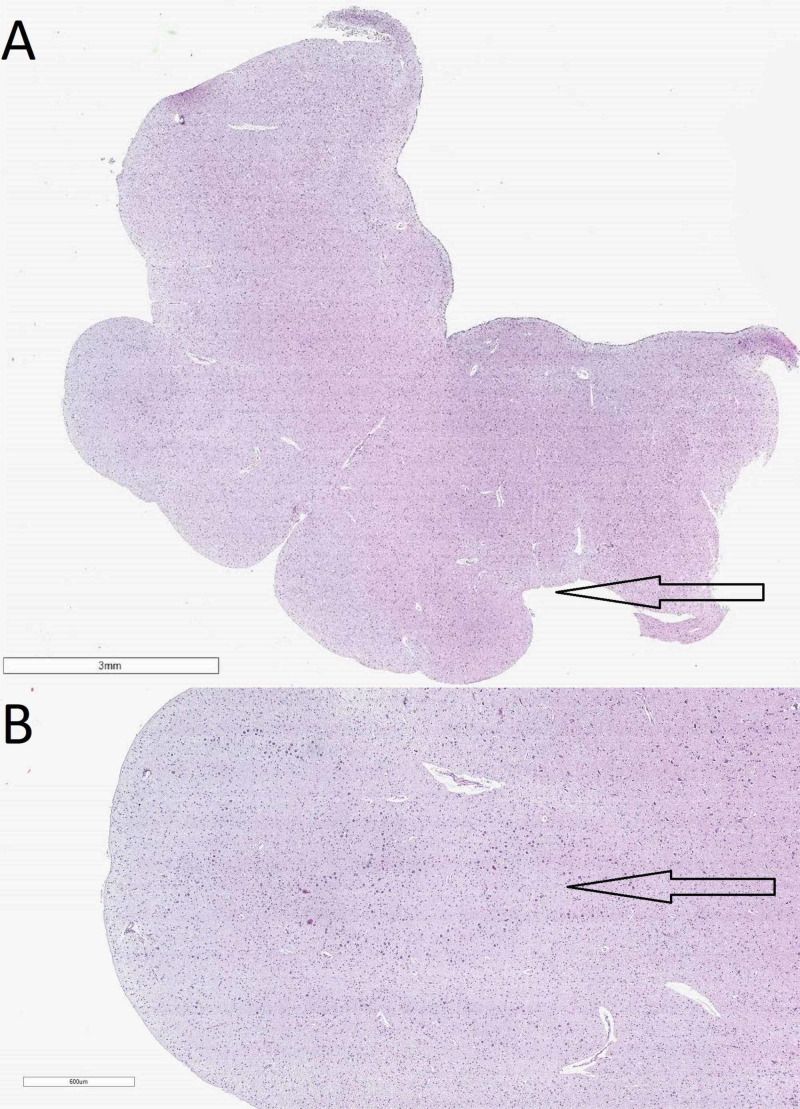
Histology from the medulla oblongata A: macro slide view of the medulla oblongata with a severely hypoplastic spinocerebellar tract (arrow) (hematoxylin and eosin stain); B: decreased neuronal number in the olivary nuclei (arrow) (original magnification x40)

**Figure 6:**
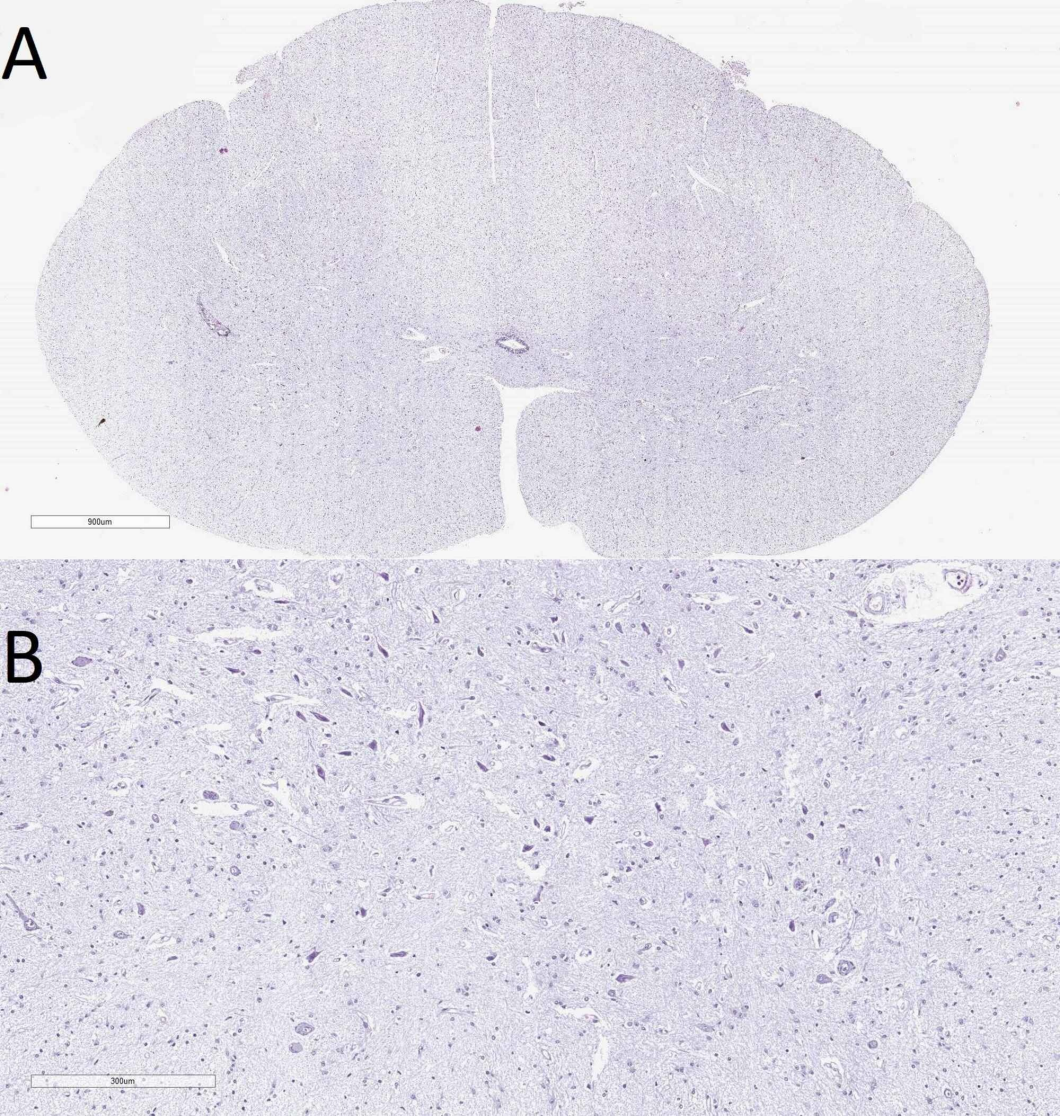
Histology of the spinal cord A: macro slide view from the spinal cord (hematoxylin and eosin stain); B: decreased neuronal number in the anterior horns with degenerative neurons (original magnification x100)

The medulla oblongata revealed a narrowed central canal, decreased neuronal count in the olivary nuclei as well as severely hypoplastic spinocerebellar tract (Figure 5). The spinal cord also showed decreased neuronal count in the anterior horns and central canal stenosis (Figure 6).

The clinical manifestation of hypoventilation respiratory failure with a right-to-left cardiac shunt and the gross and morphological changes in the cerebellum, brainstem, and spinal cord, with combined microcephaly and internal hydrocephalus, were in concordance with the morphological substrate of PCH type 1B. Therefore, the protocol was finalized as PCH type 1B to be the main condition.

## Discussion

PCHs are genetic diseases with recessive inheritance; having blood-relative parents, as in our case, severely increases the chance of their occurrence [6]. All such pregnancies should be extensively monitored and, if malformations of the CNS are noted, the pregnancy could be terminated. Even in unfollowed pregnancies, children from blood relatives, especially those with malformative stigmata and neurological symptoms, as in our case, should be subjected to CNS imaging: transfontanelle echography, CT, or MRI, and extensive investigation of the internal organs, especially the heart. Furthermore, if there are other children from the same parents, they should also be subjected to extensive clinical tests, to rule out not only less severe forms of PCH type 1B but other malformations or syndromes as well [6,7].

Based on the severity of the findings in our case, the development of pneumonia is highly likely; however, inflammation was not observed in the lungs. This could be attributed both to a nearly two-week antibiotic therapy as well as to severe atelectasis mimicking the clinical and radiological findings of diffuse pneumonia. Clinically, it has been reported that patients with PCH type 1B can have a more favorable prognosis than in our case; however, the presence of respiratory complications, mainly hypoventilation pneumonia, is most often the cause of death in the pediatric population [2,3,7,8]. It is highly unlikely that the acute respiratory infection in the seventh month of gestation is a contributing factor to the development of PCH, as the cerebellum is already formed by the seventh month of gestation and infections affecting the CNS develop in earlier months of gestation and have a more generalized and pronounced clinical manifestation.

## Conclusions

PCH type 1B is a rare autosomal recessive disease characterized by gross and cellular signs of hypoplasia in the cerebellum, brainstem, and spinal cord. Clinically, it presents mainly with respiratory failure and muscle weakness. Our case highlights the gross and histological findings to be taken into consideration on autopsy in the presence of gross findings and lack of any clinical suspicions.
